# Fat extract improves fat graft survival via proangiogenic, anti-apoptotic and pro-proliferative activities

**DOI:** 10.1186/s13287-019-1290-1

**Published:** 2019-06-13

**Authors:** Hongjie Zheng, Ziyou Yu, Mingwu Deng, Yizuo Cai, Xiangsheng Wang, Yuda Xu, Lu Zhang, Wenjie Zhang, Wei Li

**Affiliations:** Department of Plastic and Reconstructive Surgery, Shanghai Key Laboratory of Tissue Engineering, Shanghai 9th People’s Hospital, Shanghai Jiao Tong University, 639 Zhi Zao Ju Road, Shanghai, 200011 China

**Keywords:** Fat extract, Nanofat, Fat transplantation, Proangiogenic, Anti-apoptotic, Pro-proliferative

## Abstract

**Background:**

Our previous study proved that nanofat could enhance fat graft survival by promoting neovascularization. Fat extract (FE), a cell-free component derived from nanofat, also possesses proangiogenic activity.

**Objectives:**

The aim of this study was to investigate whether FE could improve fat graft survival and whether FE and nanofat could work synergistically to promote fat graft survival. The underlying mechanism was also investigated.

**Methods:**

In the first animal study, human macrofat from lipoaspirate was co-transplanted into nude mice with FE or nanofat. The grafts were evaluated at 2, 4 and 12 weeks post-transplantation. In the second animal study, nude mice were transplanted with a mixture of macrofat and nanofat, followed by intra-graft injection of FE at days 1, 7, 14, 21 and 28 post-transplantation. The grafts were evaluated at 12 weeks post-transplantation. To detect the mechanism by which FE impacts graft survival, the proangiogenic, anti-apoptotic and pro-proliferative activities of FE were analysed in grafts in vivo and in cultured human vascular endothelial cells (HUVECs), adipose-derived stem cells (ADSCs) and fat tissue in vitro.

**Results:**

In the first animal study, the weights of the fat grafts in the nanofat- and FE-treated groups were significantly higher than those of the fat grafts in the control group. In addition, higher fat integrity, more viable adipocytes, more CD31-positive blood vessels, fewer apoptotic cells and more Ki67-positive proliferating cells were observed in the nanofat- and FE-treated groups. In the second animal study, the weights of the fat grafts in the nanofat+FE group were significantly higher than those of the fat grafts in the control group. In vitro, FE showed proangiogenic effects on HUVECs, anti-apoptotic effects on fat tissue cultured under hypoxic conditions and an ability to promote ADSC proliferation and maintain their multiple differentiation capacity.

**Conclusions:**

FE could improve fat graft survival via proangiogenic, anti-apoptotic and pro-proliferative effects on ADSCs. FE plus nanofat-assisted fat grafting is a new strategy that could potentially be used in clinical applications.

## Background

Soft tissue defects caused by congenital defects, trauma, tumour resection and ageing are the most common problems in plastic and reconstructive surgery. Traditional repairs include filling with artificial fillers and autologous tissue transplantation. The potential rejection reactions induced by artificial fillers limit their clinical application. With biocompatibility and a wide range of sources, autologous fat grafting has become one of the most commonly used methods for the treatment of soft tissue defects. However, the highly unpredictable absorption rate of grafted fats after fat transplantation reduces the efficacy of this method. The retention rate of grafted fats is reported to range from 20 to 80% [1–4].

To address this issue, numerous attempts have been made to increase fat graft survival. In the early stage after fat grafting, adipocytes obtain nutrients and oxygen through plasmatic diffusion from the surrounding tissues, and this process is limited to the peripheral zone of the grafted fat. In Hitomi Eto’s research, only peripheral adipocytes survived, and the depth of the viable zone was approximately 300 μm from the graft edge; almost all adipocytes located deeper than 300 μm died within a few days after grafting [5]. Therefore, early revascularization of the grafted fat is crucial for survival, especially in patients who undergo large-volume fat grafting [5–10]. Co-transplantation of fat with proangiogenic growth factors (vascular endothelial growth factor (VEGF), platelet-derived growth factor (PDGF), basic fibroblast growth factor (bFGF), etc.) has been proven to enhance the fat graft retention rate by increasing revascularization. In 2006, Matsumoto et al. demonstrated that the stromal vascular fraction (SVF) isolated from fat tissues could improve the survival of fat grafts, and a cell-assisted lipotransfer strategy has been developed [11]. In recent years, adipose-derived stem cells (ADSCs) have been shown to improve survival by paracrine secretion of many kinds of growth factors and direct differentiation into adipocytes and endothelial cells [12–15]. However, enzymatic digestion during SVF isolation or ADSC culture raises safety concerns.

In 2013, Tonnard et al. first described the concept of nanofat, a liquid suspension derived from fat tissue that is obtained by mechanical emulsification and filtering, and showed remarkable improvements in skin quality [16]. Subsequent studies revealed that nanofat also has promising effects on skin rejuvenation, scar management and reconstruction of tissue defects [17–19]. Nanofat contains cellular and non-cellular components. It is processed simply by mechanical means without any chemicals or enzymes [16]. Therefore, nanofat is much safer and more time saving for clinical applications than SVF and ADSCs. In a previous study, we found that compared to the control group, co-transplantation of fat with nanofat yielded a higher graft retention rate, better histological structure and higher capillary density of grafts [20]. Subsequent assays have demonstrated that nanofat contains a large number of cytokines and growth factors (VEGF, transforming growth factor beta (TGF-β), bFGF, hepatocyte growth factor (HGF), granulocyte-macrophage colony-stimulating factor (GM-CSF), insulin-like growth factor 1 (IGF-1) and PDGF), which may account for the improvement of fat graft survival [20].

Fat extract (FE), the liquid fraction that is derived from nanofat using a mechanical approach to remove the cellular components and the lipid remnants, was first described in our previous study and showed proangiogenic effects in a murine model of limb ischaemia [21]. FE is a cell-free fraction that could avoid potential cell-related concerns in clinical applications, such as genetic mutations, and the difficulties of long-term storage. In the present study, we aimed to evaluate the effects of FE on fat transplantation, to investigate the underlying mechanisms and to establish a new FE-assisted fat graft strategy for future clinical application.

## Methods

### Nanofat and FE preparation

After informed consent was obtained, lipoaspirate was obtained from the abdomen or thighs of five donors via standard vacuum-assisted liposuction from December 2017 to November 2018. All donors were healthy females with no underlying disease or diabetes mellitus. The mean body mass index was 24.86, and the mean age was 28 years (range 21–40 years). The study was approved by the Ethics Committee of Shanghai Jiaotong University School of Medicine, Shanghai, China. Macrofat was harvested via a standard 3-mm liposuction cannula with large side holes 2 × 7 mm [16]. The lipoaspirate was allowed to settle for 15 min to allow the layers to separate by gravity sedimentation, and the superior oil and inferior blood layers were then removed by vacuum aspiration. The remaining fat layer was subsequently rinsed with saline 3 times in closed syringes and then centrifuged at 1200 rpm for 4 min at 4 °C. Any oil and fluid were removed again, and the remaining fat was collected for further processing.

Nanofat was obtained using Tonnard’s technique [16]. Briefly, the nanofat was obtained by mechanical emulsification; the macrofat was transferred between two 10-cc syringes connected by a female-to-female Luer-Lok connector 60 times (B. Braun Medical Inc., Melsungen, Germany). FE was produced from nanofat by centrifugation, as previously described [21]. Briefly, the nanofat was centrifuged at 1200 rpm for 5 min, and the fat was separated into four layers. The third aqueous layer, namely, FE, was collected and filtered through a 0.22-μm filter (Corning Glass Works, Corning, NY, USA) for further use.

### Mouse fat graft model

All experiments were approved by the Animal Care and Experiment Committee of Shanghai Jiaotong University School of Medicine. Six-week-old male BALB/c-nu nude mice (Animal Laboratory, The Ninth People’s Hospital, Shanghai Jiaotong University School of Medicine, Shanghai, China) were used in this study.

**Fig. 1:**
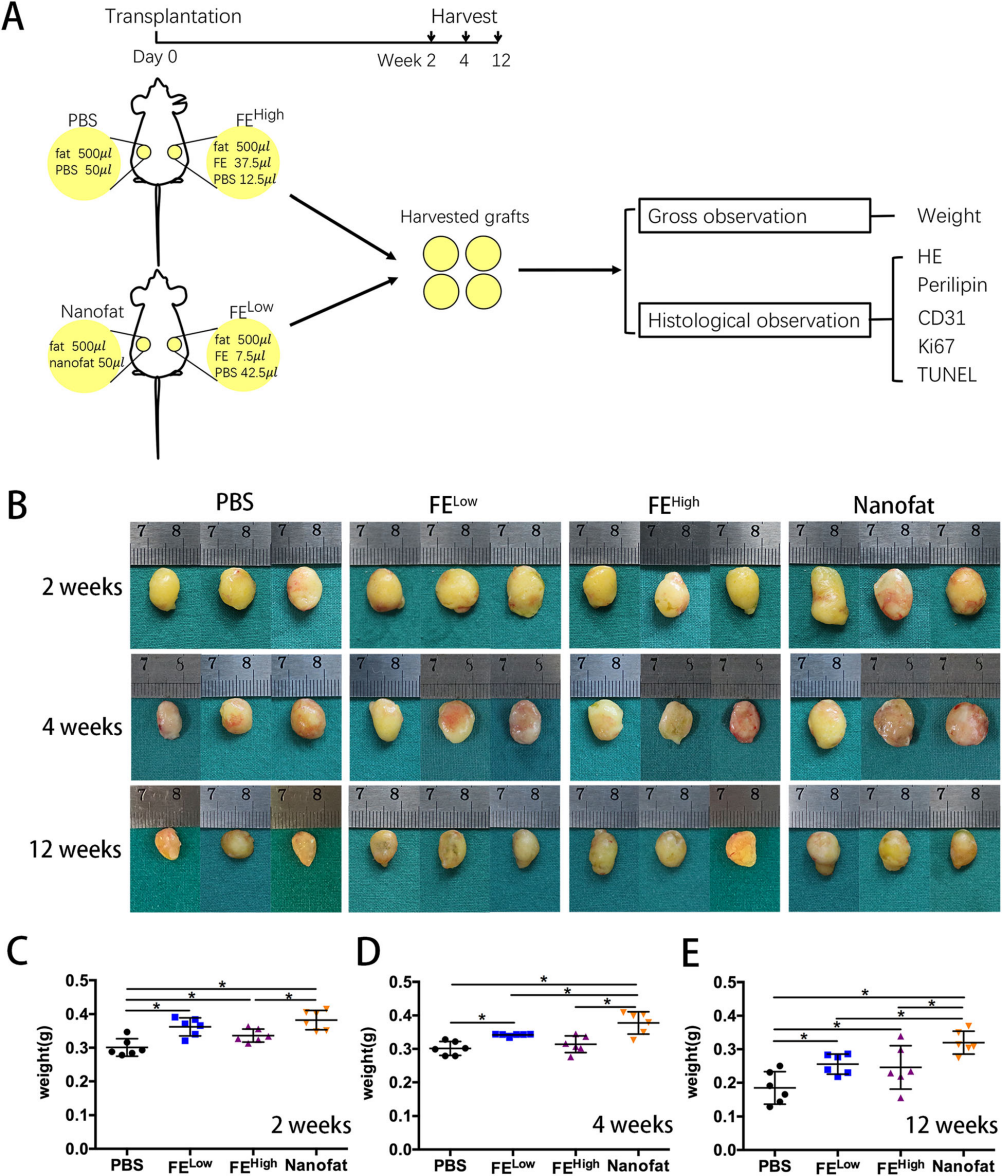
FE and nanofat improved fat graft survival. **a** The design of the first animal study. **b** Macroscopic images of the fat grafts at 2, 4 and 12 weeks post-transplantation. **c** Weights of the fat grafts at 2 weeks post-transplantation (*p < 0.05). **d** Weights of the fat grafts at 4 weeks post-transplantation (*p < 0.05). **e** Weights of the fat grafts at 12 weeks post-transplantation (*p < 0.05)

To determine the effects and investigate the mechanisms of FE on fat transplantation, four groups were included in the first experiment (Fig. 1a): the phosphate-buffered saline (PBS) group, the FE^Low^ group, the FE^High^ group and the nanofat group. In the PBS group, a mixture of 500 μl of fat with 50 μl of PBS was injected. In the nanofat group, a mixture of 500 μl of fat with 50 μl of nanofat was injected. In the FE^Low^ group, 500 μl of fat was injected with 7.5 μl of FE and 42.5 μl of PBS, and the FE^High^ group received 500 μl of fat injected together with 37.5 μl of FE and 12.5 μl of PBS. The total volume of each graft was 550 μl. The average output ratio from nanofat to FE is 15%; we added 7.5 μl of FE in the FE^Low^ group which was equivalent to 50 μl of nanofat in the nanofat group. The amount of FE in the FE^High^ group was 5 times that of the FE^Low^ group. Thirty-six mice were used in this experiment, and the injection was performed in the dorsal region of nude mice (each mouse was injected at two sites) via 14-G needles, as indicated in Fig. 1a. Mice were sacrificed at 2, 4 and 12 weeks post-transplantation, and the fat grafts were harvested and weighed (*n* = 6).

To determine whether the nanofat and FE could work synergistically to promote fat graft survival, a second experiment was carried out as described in Fig. 7a. Twelve mice were used, and each animal received two grafts (a mixture of 500 μl of fat together with 50 μl of nanofat) on its back. The graft on the left side of the back underwent PBS injection, and the graft on the right side of the back underwent FE injection at days 1, 7, 14, 21 and 28 post-transplantation. The injection was performed at 4 points around the grafted fat and 1 point over the graft (10 μl each point). The grafted fat was harvested and weighed at 12 weeks post-transplantation.

### Histological staining

Fat grafts were fixed in 4% paraformaldehyde and embedded in paraffin. The samples were then sectioned and stained with haematoxylin-eosin (HE). Fat integrity, vacuoles and fibrosis were evaluated as described by Hu et al. [22]. For immunohistological staining, paraffin-embedded tissue sections were incubated with rabbit anti-CD31, anti-Ki67 and anti-perilipin antibodies (Abcam, Cambridge, UK), followed by incubation with a horseradish peroxidase-conjugated secondary antibody (Dako Glostrup, Denmark) or Alexa Fluor 488-conjugated secondary antibodies (Invitrogen, California, USA). CD31-positive blood vessels, Ki67-positive proliferating cells and the percentage area of perilipin-positive adipocytes were calculated. To analyse cell apoptosis, terminal deoxynucleotidyl transferase-mediated d-UTP nick end labelling (TUNEL) staining was performed according to the manufacturer’s instructions, and apoptotic cell numbers were calculated. Five random fields were selected for each sample (*n* = 3/group). All measurements were performed with Image-Pro Plus software (Media Cybernetics; Silver Spring, MD, USA).

### Fat tissue culture and measurement of viable adipocytes

The lipoaspirate was divided into four groups as above: the PBS group, the FE^Low^ group, the FE^High^ group and the nanofat group. Fat tissue for each group was cultured in an incubator under hypoxic (1% oxygen) conditions. The fat tissue was then examined by immunostaining with perilipin to detect viable adipocytes at the beginning of tissue culture (day 0) and 24 h later (day 1).

### Cell culture

For primary culture of ADSCs, the lipoaspirate was digested with 0.3% collagenase at 37 °C for 1 h and then centrifuged at 1500 rpm for 5 min. The cell pellet was resuspended in Dulbecco’s modified Eagle’s medium (DMEM) (HyClone) containing 10% fetal bovine serum (FBS) and 1% penicillin-streptomycin antibiotic (Gibco, Carlsbad, CA, USA) and then seeded in a cultured dish. Cell passaging was performed until the cells reached 80–90% confluence. Upon reaching passage 2, FE at different concentrations (0, 1, 2 and 5%) was added to the cell culture supplements to evaluate the effects of FE on ADSCs. Cell numbers were counted at each passage to evaluate the pro-proliferative effect of FE on ADSCs. Characterization of ADSCs was performed when the cells reached passage 5.

Human vascular endothelial cells (HUVECs) were purchased from the American Type Culture Collection (ATCC, Rockville, MD, USA) and were maintained in DMEM supplemented with 10% FBS and 1% penicillin-streptomycin antibiotic. The culture medium was changed every 2 to 3 days, and the culture was maintained at 37 °C in a humidified atmosphere of 95% air and 5% CO_2_.

### Characterization of ADSCs

#### Trilineage differentiation of ADSCs

The trilineage differentiation potential of ADSCs was measured as previously described [23]. All chemicals were purchased from Sigma (St. Louis, MO, USA) unless otherwise stated. For adipogenic differentiation, ADSCs were cultured in adipogenic induction medium (penicillin/streptomycin, glutamine, 10% FBS, insulin, dexamethasone, 3-isobutyl-methylxanthine and rosiglitazone) for 21 days, followed by Oil Red O staining. For osteogenic differentiation, ADSCs were cultured for 21 days in an osteogenic induction medium (penicillin/streptomycin, glutamine, 10% FBS, dexamethasone, β-glycerophosphate and ascorbic acid) and then assayed by Alizarin Red staining. For chondrogenic differentiation, 5 × 10^5^ ADSCs were pelleted by centrifugation in a 15-ml culture tube. The pelleted ADSCs were then cultured in chondrogenic induction medium using Chondro BulletKit (Lonza Walkersville Inc., Walkersville, MD, USA). After 21 days, the pellet was fixed in 4% paraformaldehyde, embedded in paraffin, and sectioned and stained with Alcian Blue solution.

#### Phenotypic profile of ADSCs

The phenotypic profile of ADSCs was determined by flow cytometry as previously described [24, 25]. Briefly, the cells were collected; stained with phycoerythrin (PE)-conjugated anti-CD11b, anti-CD19, anti-CD34, anti-CD45, anti-HLA-DR, anti-CD73, anti-CD90 and anti-CD105 antibodies (BD Bioscience, San Jose, CA); and analysed using an Epics Altra flow cytometer (Beckman Coulter, CA, USA).

### Cell proliferation assay

HUVECs were seeded in a 96-well plate at 1 × 10^3^ cells per well and cultured in DMEM medium containing 10% FBS for 24 h. The cells were then treated for 3 days with different concentrations of FE (0, 1, 2 and 5%). Untreated cells were used as a control. Cell Counting Kit-8 (CCK-8; Dojindo Molecular Technologies, Rockville, MD, USA) was used for the cell proliferation assay. The absorbance spectrum at 450 nm was recorded using a microplate reader (SpectraMAX i3x; Molecular Devices, Sunnyvale, CA, USA).

### Cell migration assay

HUVECs were seeded in 6-well plates and grown to a monolayer. Wounds were created by scratching with a sterile pipette tip, and the medium was replaced with DMEM supplemented with 2% FBS. FE (0, 1, 2, 5%) was added to the medium. Images were captured with a digital camera at 0 and 24 h after scratching and measured with ImageJ software (NIH, Bethesda, MD, USA). The data are reported as the relative percentage of wounds healed.

### Tube formation assay

A tube formation assay was performed in Matrigel (BD Biosciences). HUVECs were suspended in DMEM supplemented with 2% FBS. HUVECs (1.5 × 10^4^) were seeded onto Matrigel-coated 96-well plates. The plate was incubated at 37 °C in 5% CO_2_ for 6 h. The cells were then stained with Calcein-AM solution (Yeason, Shanghai, China). Tube formation was photographed under a fluorescence microscope (Carl Zeiss, Oberkochen, Germany). The number of junctions was calculated using ImageJ software (NIH).

### Proteinase K or RNase treatment to investigate the proangiogenic activity of FE

To investigate whether the protein or ribonucleic acid (RNA) component is essential for the proangiogenic effect of FE, FE was digested with proteinase K (PK) (20 μg/ml) or ribonuclease (RNase) (200 μg/ml) at 37 °C for 1 h and then incubated with HUVECs at a concentration of 5%. Cell proliferation assays, migration assays and tube formation assays were performed as described above.

### Statistical analysis

Data are presented as the mean ± standard deviation. Differences between the groups were analysed using one-way analysis of variance (ANOVA) or non-parametric tests. Statistical analyses were performed using IBM SPSS software (SPSS Statistics V22, IBM Corporation, USA). Values of *p* < 0.05 were considered statistically significant.

## Results

### FE and nanofat improved fat graft survival

To determine the effects of FE on fat transplantation, human fat tissues were transplanted into nude mice together with PBS, nanofat or FE (Fig. 1a). The fat grafts in each group were harvested at 2, 4 and 12 weeks post-transplantation (*n* = 6/time point/group). Representative grafts in each group are shown in Fig. 1b. A decrease in graft size from 2 to 12 weeks was observed in all groups, which was confirmed by measuring the weight of each fat graft (Fig. 1c–e). The weights of the fat grafts in the nanofat group and the FE-treated groups were significantly (*p* < 0.05) higher than those in the PBS group. In addition, the weights of the fat grafts in the nanofat group were significantly (*p* < 0.05) higher than those in the FE-treated groups. No significant difference was observed between the FE^Low^ and FE^High^ groups.

### Histological evaluation of grafts

**Fig. 2:**
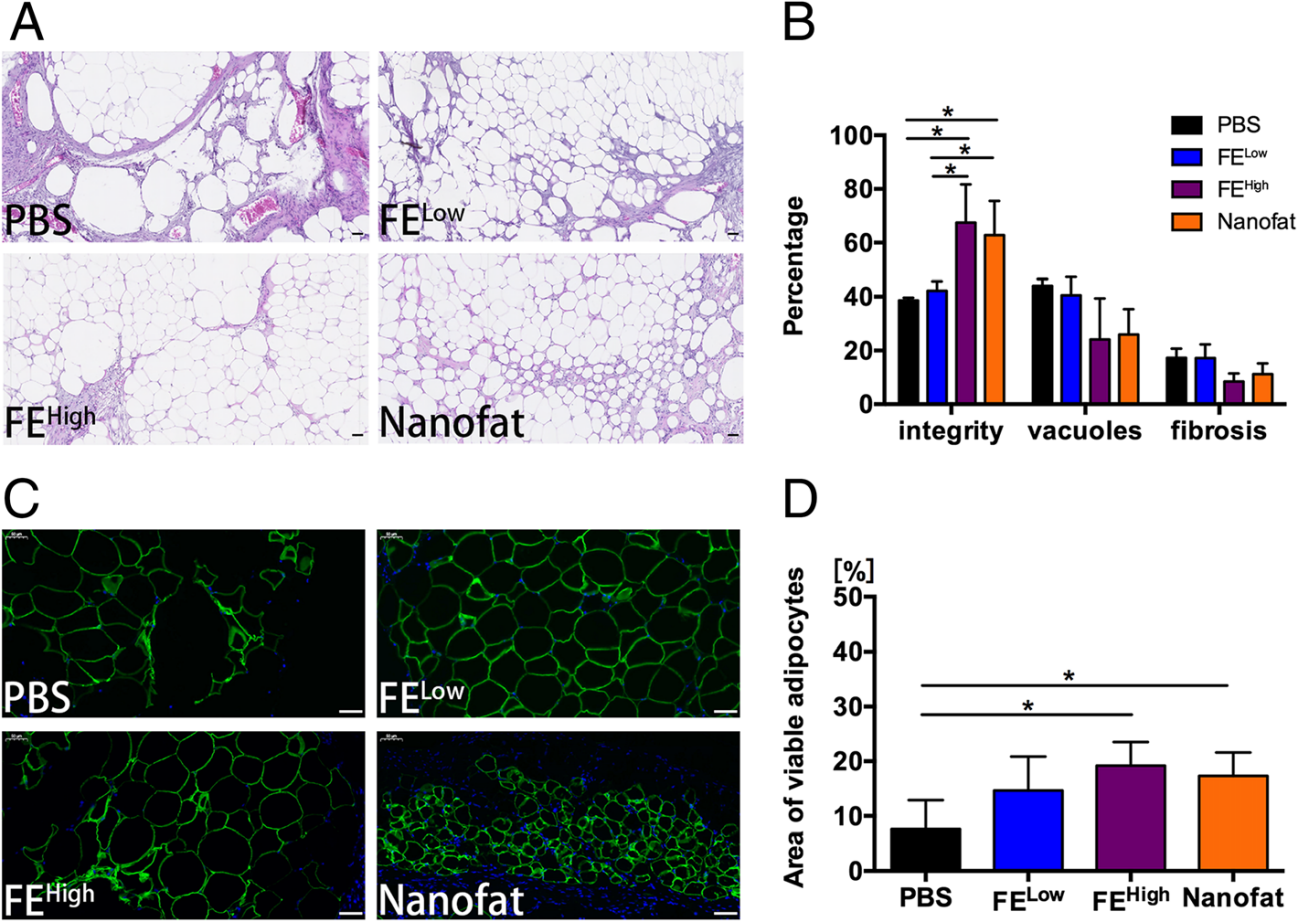
Histological evaluation of grafts. **a** HE staining of the harvested fat grafts at 12 weeks post-transplantation. Scale bars = 100 μm. **b** Histologic analysis of the grafts at 12 weeks post-transplantation. The percentage areas of integrity, vacuoles and fibrosis were calculated (*p < 0.05). **c** Perilipin staining of the harvested fat grafts at 12 weeks post-transplantation. Scale bars = 50 μm. **d** The percentage area of viable adipocytes in the grafts at 12 weeks post-transplantation was calculated (*p < 0.05)

At 12 weeks post-transplantation, the grafts were harvested for histological analyses. After HE staining, more small adipocytes were observed in the FE^High^ group and the nanofat group (Fig. 2a). Statistical analyses of fat integrity, vacuoles and fibrosis confirmed that higher fat integrity with fewer vacuoles and less fibrosis was observed in the FE^High^ group and the nanofat group than in the PBS group and the FE^Low^ group (Fig. 2b). The viability of adipocytes in each sample was further analysed by perilipin staining. As shown in Fig. 2c, more perilipin-positive viable adipocytes were observed in the FE^High^ group and the nanofat group, and significant differences were observed between these two groups and the PBS group (Fig. 2d). These results suggest that FE and nanofat could improve fat graft survival.

### Proangiogenic, anti-apoptotic and pro-proliferative effects of FE in vivo

**Fig. 3:**
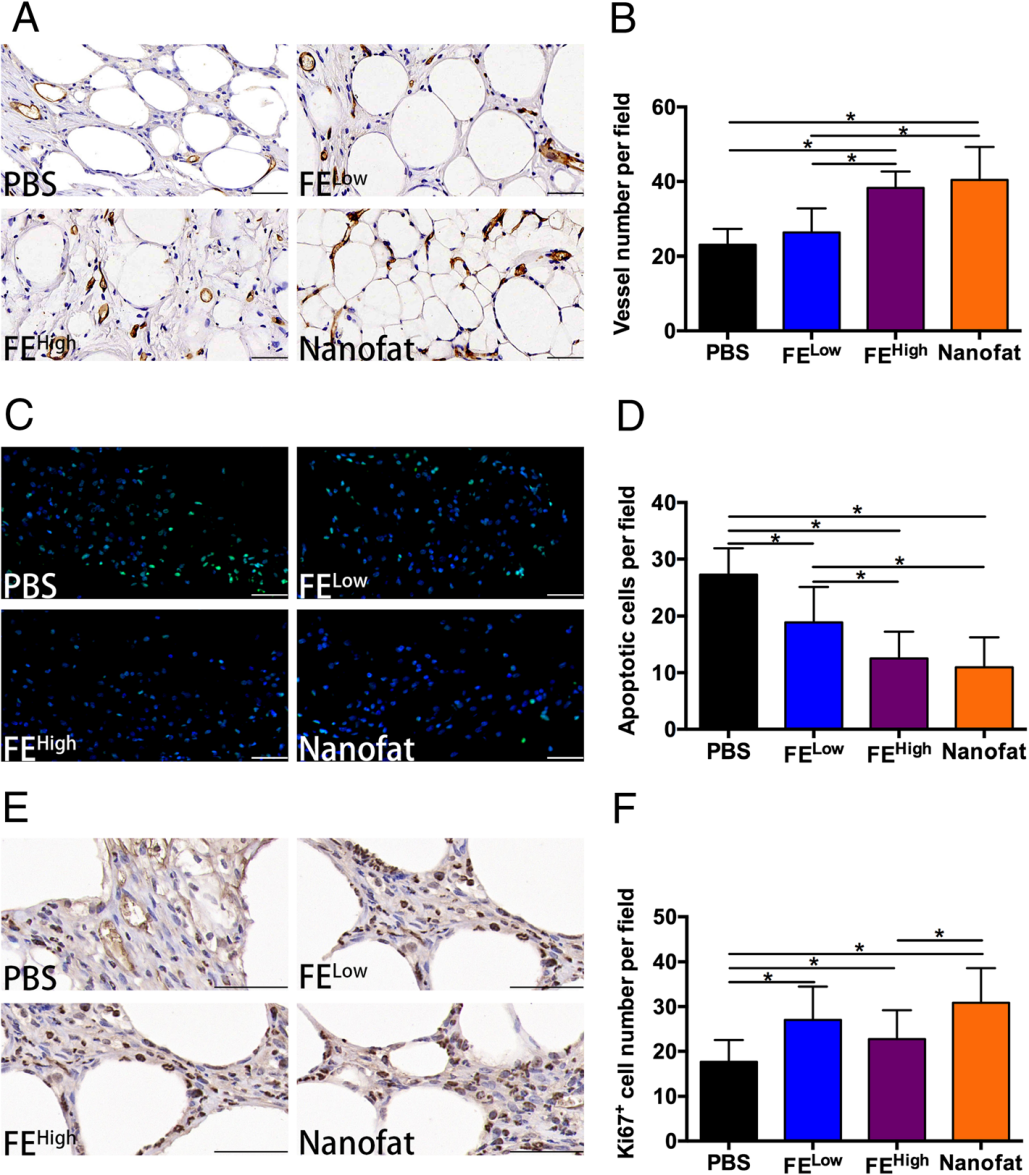
Proangiogenic, anti-apoptotic and pro-proliferative effects of FE on fat grafts*.* **a** Anti-CD31 staining of grafts at 12 weeks post-transplantation. Scale bars = 50 μm. **b** The number of CD31-positive vessels per field in the grafts (*p < 0.05). **c** TUNEL analysis of grafts harvested at 2 weeks post-transplantation. Scale bars = 50 μm. **d** The number of apoptotic cells per field in the grafts (*p < 0.05). **e** Anti-Ki67 staining of grafts at 2 weeks post-transplantation. Scale bars = 50 μm. **f** The number of Ki67-positive cells per field in the grafts (*p < 0.05)

To investigate the mechanisms of FE in fat grafting, grafts at 12 weeks post-transplantation were harvested for anti-CD31 staining, and grafts at 2 weeks post-transplantation were harvested for TUNEL and anti-Ki67 staining. The number of CD31-positive blood vessels per field in the FE^High^ group (38.29 ± 4.06) and the nanofat group (40.41 ± 8.61) was significantly higher than that in the PBS group (22.96 ± 4.16) and the FE^Low^ group (26.32 ± 6.28) (*p* < 0.05). No significant difference was observed between the FE^High^ group and the nanofat group (Fig. 3a, b), indicating that FE and nanofat could promote neovascularization.

TUNEL staining showed that the average number of apoptotic cells per field in the PBS, the FE^Low^, the FE^High^ and the nanofat groups were 27.25 ± 4.53, 18.89 ± 6.08, 12.52 ± 4.64 and 10.96 ± 5.20, respectively. There were significantly fewer apoptotic cells in the FE and nanofat groups than in the PBS group (Fig. 3c, d), indicating that FE and nanofat might improve graft survival through their anti-apoptotic activity.

The proliferating cells in the grafts were analysed by anti-Ki67 staining. As shown in Fig. 3 e and f, the numbers of Ki67-positive proliferating cells in the PBS, FE^Low^, FE^High^ and nanofat groups were 17.67 ± 4.69, 27.00 ± 7.23, 22.73 ± 6.22 and 29.00 ± 6.35 cells per field, respectively. The numbers of proliferating cells in the nanofat and FE groups were significantly higher than those in the PBS group*.*

### Proangiogenic, anti-apoptotic and pro-proliferative effects of FE in vitro

The proangiogenic, anti-apoptotic and pro-proliferative effects of FE were further confirmed by measuring the effects of FE on cultured vascular endothelial cells, ADSCs and fat tissue in vitro.

**Fig. 4:**
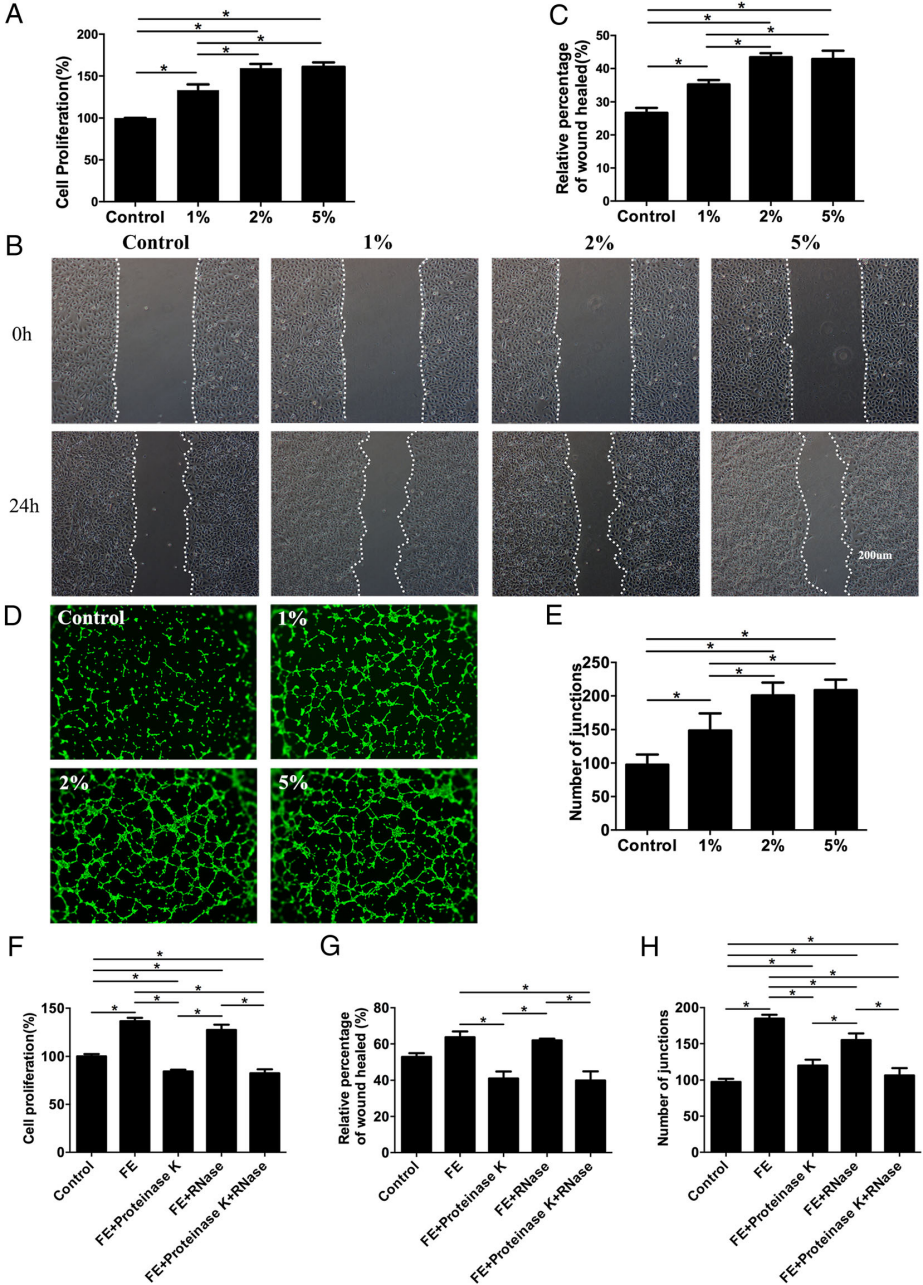
FE promotes endothelial cell proliferation, migration and tube formation. **a** Cell proliferation was assessed using a cell counting kit, and the percentage of optical density values relative to the control was calculated (*p < 0.05). **b** HUVEC migration was evaluated using a cell migration assay. **c** The relative percentage of wound healing (24 h) was quantified (*p < 0.05). **d** Tube formation of HUVECs was performed on solidified Matrigel and stained using Calcein-AM. **e** The assessment of the number of junctions/mm^2^ in each group is shown (*p < 0.05). **f**–**h** FE was digested with PK or RNase before incubation with HUVECs. **f** Cell proliferation was assessed using a cell counting kit, and the percentage of optical density values relative to the control was calculated (*p < 0.05). **g** The relative percentage of wound healing (24 h) was quantified (*p < 0.05). **h** The assessment of the number of junctions/mm^2^ in each group is shown (*p < 0.05)

To examine the proangiogenic ability, the effects of FE on HUVEC proliferation, migration and tube formation were evaluated. Cell proliferation assays showed that FE promoted HUVEC growth in a dose-dependent manner (Fig. 4a). Cell migration activity analysed by a wound healing assay also showed that FE enhanced the migration of HUVECs in a dose-dependent manner (Fig. 4b, c). An in vitro tube formation assay revealed that more vascular-like structures were formed in the FE-treated groups (Fig. 4d), and the number of junctions was significantly increased in the FE-treated groups (Fig. 4e). FE contains proteins and RNAs. To clarify which component is essential for proangiogenic activity, FE was digested with PK or RNase (a broad-specificity protease or RNase) before incubation with HUVECs. Interestingly, the cell proliferation, migration and tube formation of HUVECs were reduced by PK treatment alone or by PK and RNase co-treatment but not by RNase treatment (Fig. 4f–h), indicating that the proteins in FE are essential for the observed proangiogenic activity.

**Fig. 5:**
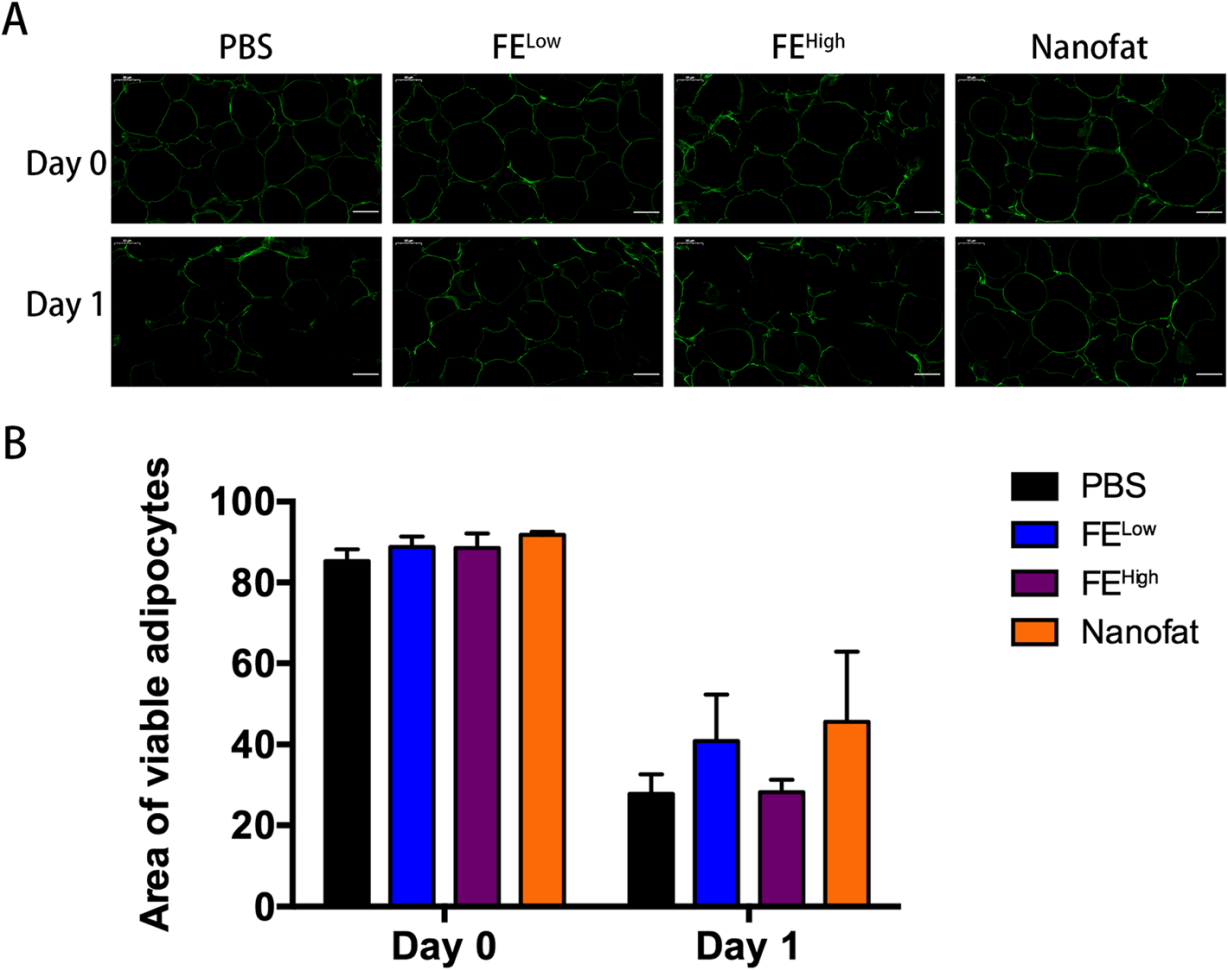
The anti-apoptotic effects of FE on fat tissues cultured under hypoxic conditions. **a** Immunofluorescence analysis of adipocyte viability in fat tissue cultured under hypoxic conditions. Scale bars = 50 μm. **b** The percentage area of viable adipocytes of fat tissue at different time points was calculated (*p < 0.05)

The anti-apoptotic activity of FE was evaluated by culturing fat tissues under hypoxic conditions in the presence and absence of FE. As shown in Fig. 5, at the beginning of the tissue culture (day 0), no difference in the viable cell area was observed between the control group and the experimental groups. However, 24 h after culture, the adipocyte viability in the FE^Low^ and nanofat groups was higher than that in the PBS group, but no statistically significant difference (*p* > 0.05) was observed.

**Fig. 6:**
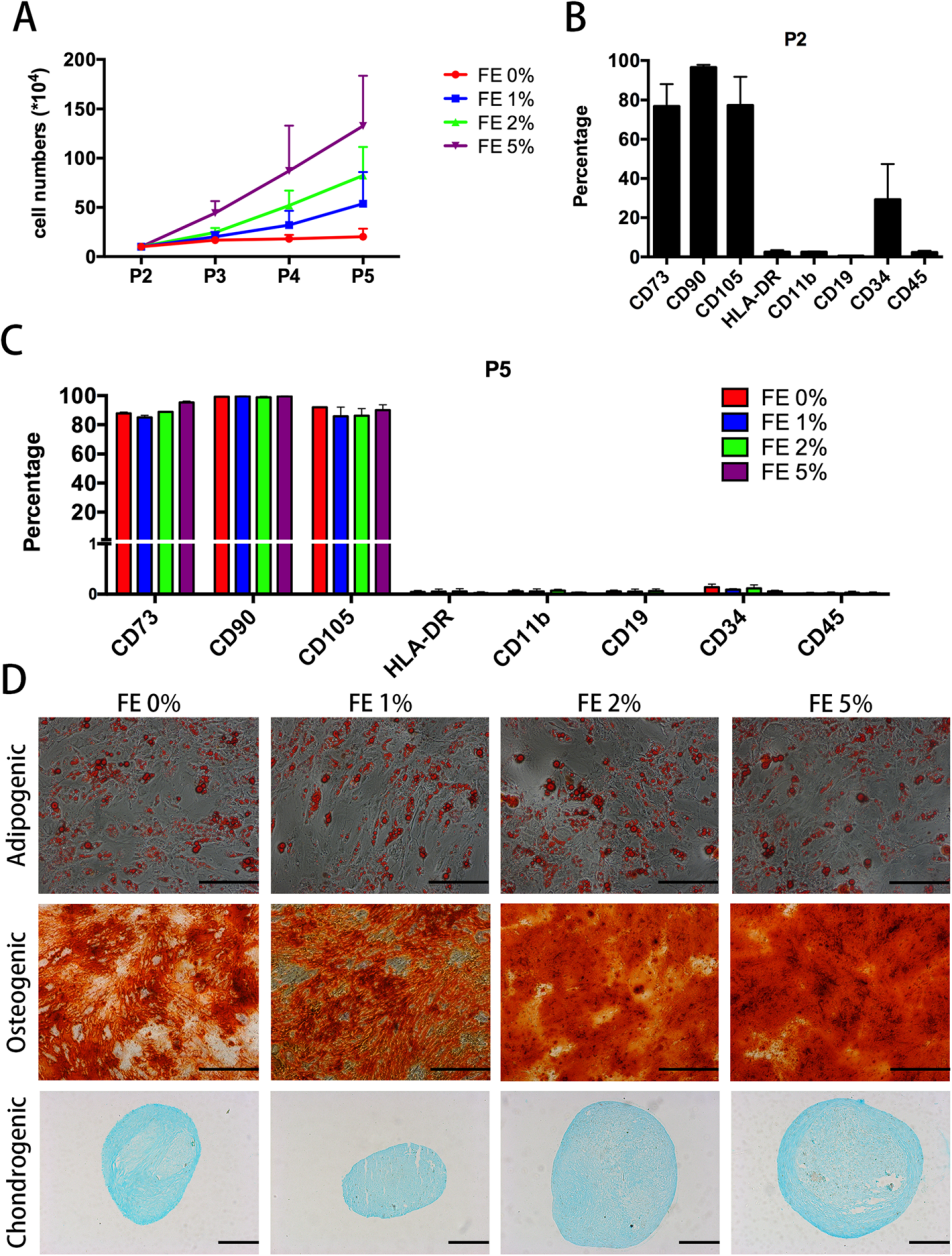
FE promotes ADSC proliferation and maintains their multipotent differentiation capacity. **a** ADSCs were treated with FE at the indicated concentrations. Cell proliferation was assessed by counting the cell numbers at each passage. **b** The cell surface marker expression of ADSCs at passage 2 was analysed. **c** The cell surface marker expression of ADSCs at passage 5 was analysed. **d** The differentiation capacity of human ADSCs into the adipogenic, osteogenic and chondrogenic lineages was analysed. Differentiation was detected with Oil Red O, von Kossa and Alcian Blue staining. Scale bars = 100 μm

To determine the effect of FE on ADSCs, cells were passaged with different concentrations of FE from passage 2 to passage 5. Cell counts showed that the cells proliferated more robustly in the presence of FE, with dose-dependent effects (Fig. 6a). Whether the cells that expanded in the presence of FE lost their “stemness”, cell surface marker expression and differentiation capacity at passage 5 were analysed. As shown in Fig. 6b and c, ADSCs at passage 2 were positive for CD73, CD90 and CD105 and negative for CD11b, CD19, CD34, CD45 and HLA-DR. Increased levels of CD73, CD90 and CD105 expression and decreased levels of CD11b, CD19, CD34, CD45 and HLA-DR expression were observed in both the FE-treated and non-treated groups. Cell differentiation assays showed that the adipogenic, osteogenic and chondrogenic differentiation capacities of the cells were preserved in the FE-treated groups at passage 5 (Fig. 6d). These results demonstrated that FE could promote ADSC proliferation and maintain their multipotent differentiation capacity.

### FE plus nanofat-assisted fat grafting

**Fig. 7:**
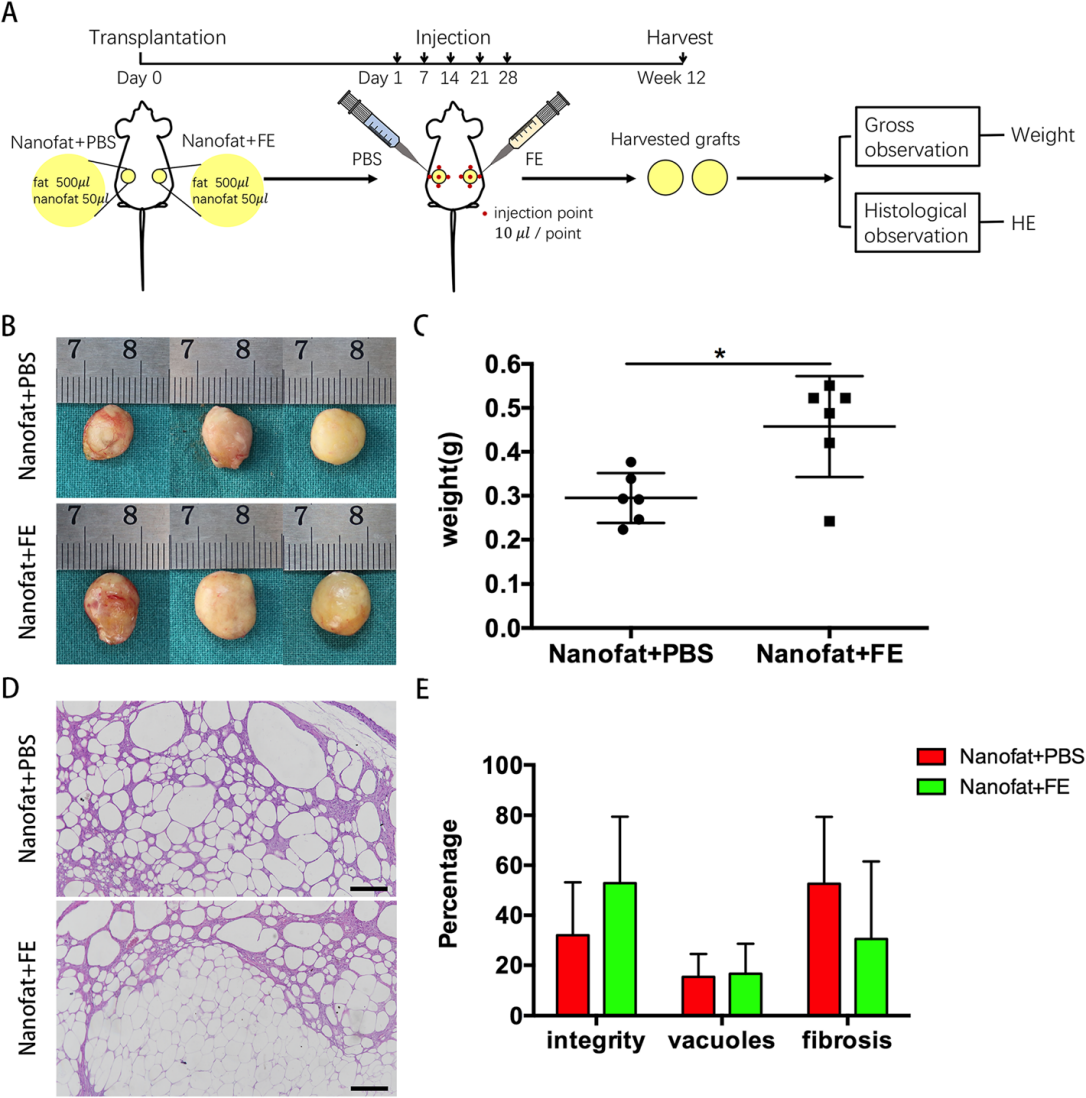
Nanofat and FE work synergistically to promote fat graft survival. **a** The design of the second animal study. **b** Macroscopic images of the fat grafts at 12 weeks post-transplantation. **c** Weights of the fat grafts at 12 weeks post-transplantation (*p < 0.05). **d** HE staining of the harvested fat grafts at 12 weeks post-transplantation. Scale bars = 100 μm. **e** Histologic analysis to evaluate the grafts at 12 weeks post-transplantation. The percentage areas of integrity, vacuoles and fibrosis were calculated (*p < 0.05)

To determine whether nanofat and FE work synergistically to promote fat graft survival, macrofat was transplanted with nanofat in nude mice, followed by intra-graft injection of FE at days 1, 7, 14, 21 and 28 post-transplantation (Fig. 7a). The grafts harvested at 12 weeks post-transplantation showed that the size of the grafts in the FE-treated group was larger than that in the PBS-treated group, which was confirmed by measuring the weights of the grafts. The weight of the nanofat+FE group (0.458 ± 0.105 g) was significantly higher than that of the nanofat+PBS group (0.295 ± 0.052 g) (*p* < 0.05) (Fig. 7b, c). Histological analysis showed higher fat integrity with less fibrosis in the nanofat+FE group than in the nanofat+PBS group. However, no significant difference was observed between the two groups (Fig. 7d, e).

## Discussion

Our previous research found that nanofat could enhance fat graft survival [20]. In the current study, we demonstrated that the cell-free FE purified from nanofat could also enhance fat graft survival. However, the effects of FE were not as strong as those of nanofat. There are two accepted theories on how fat grafts survive after transplantation, the “graft survival theory” and the “fat regeneration theory”. The “graft survival theory”, which was established by Peer in the 1950s, states that some transplanted living adipocytes survive and remain alive for a long period of time after grafting [8, 26, 27]. The “regeneration theory” states that adipocytes die early under ischaemic conditions, whereas adipose-derived stem or progenitor cells can survive under ischaemic conditions and are activated and contribute to subsequent adipose tissue regeneration [5]. Nevertheless, vascularization is crucial for fat survival and regeneration. Our previous work showed that nanofat contains a variety of growth factors, including VEGF, TGF-β, bFGF, HGF, GM-CSF, IGF-1 and PDGF, which could enhance fat graft survival by promoting neovascularization after transplantation [20]. In addition, it has been reported that nanofat also contains ADSCs [16]. In nanofat-assisted fat grafting, live ADSCs in nanofat could survive and possibly participate in fat regeneration. Therefore, co-transplantation of fat with nanofat could achieve better outcomes than co-transplantation with an equivalent amount of cell-free FE (Fig. 1). In the current study, a decrease in graft size from 2 to 12 weeks was observed in all groups, which was consistent with other studies [28, 29]. The absorption period of dead adipocytes by macrophage phagocytosis within 3 months after transplantation accounts for the atrophy of the grafted fat [26].

Cytokines and growth factors, including brain-derived neurotrophic factor (BDNF), glial cell-derived neurotrophic factor (GDNF), TGF-β, HGF, bFGF, VEGF, PDGF, epidermal growth factor (EGF), neurotrophin-3 (NT-3) and G-CSF, have been detected in FE, and the proangiogenic effect of FE has been proven in ischaemic animal model [21]. In the current study, we also found more CD31-positive blood vessels in the FE groups than in the control group (Fig. 3a, b), indicating that FE enhances fat graft survival partially through the stimulation of new vessel formation. FE contains a variety of proteins and RNase [21]. Interestingly, when FE was pre-digested with PK or RNase, its proangiogenic activity was significantly diminished by PK treatment but not RNase treatment (Fig. 4f–h), indicating that the protein fraction of FE is essential for its proangiogenic activity. Based on the proteomic analysis in our previous study [21], we speculate that such effects are likely attributed to the presence of well-known proangiogenic factors in FE, such as VEGF, PDGF, bFGF and HGF. We also believe that no significant differences could be observed by simply blocking one or two factors. It is difficult to measure which of these factors plays a key role in FE.

It is well known that cell apoptosis and necrosis tend to occur under ischaemic conditions. Eto et al. demonstrated that adipocytes had poor tolerance to ischaemic stress compared to endothelial cells and ADSCs and underwent apoptosis and necrosis as early as 12 h under ischaemia-mimicking conditions [5]. In the current study, fewer TUNEL-positive apoptotic cells were observed in the grafts co-transplanted with FE or nanofat (Fig. 3c, d). Furthermore, in vitro culture of fat tissue under hypoxic conditions showed that more dead cells were observed after 24 h of culture in the control group, while fewer dead cells were observed in the FE- or nanofat-treated groups (Fig. 5), indicating that FE and nanofat exert anti-apoptotic effects on adipocytes. The presence of VEGF, HGF and IGF-1 may account for these results. Synergistic effects of combined gene delivery on enhanced VEGF secretion were reported to decrease cardiomyocyte apoptosis under hypoxic conditions [30]. Jang et al. reported that HGF-treated hepatocytes showed better cell viability, with increased expression of Mcl-1, an anti-apoptotic protein, and decreased expression of pro-apoptotic proteins (Bad, Bik and Bid) in HGF-treated hepatocytes [31]. It was reported that lentiviral-mediated overexpression of the IGF-1 gene in ADSCs could prolong the anti-apoptotic effects by activating the PI3K/Akt pathway [32]. The anti-apoptotic mechanism of FE is not clear. We found that FE could prevent dermal fibroblast death induced by ultraviolet irradiation by reducing intracellular reactive oxygen species accumulation (unpublished data). The antioxidant pathway may play a role in the anti-apoptotic effects of FE on adipocytes.

According to the “fat regeneration theory”, ADSCs play a significant role in adipose tissue regeneration because they can survive under ischaemic conditions and produce new adipocytes to replace dead adipocytes [5]. In the current study, more Ki67-positive proliferating cells were observed in the grafts co-transplanted with FE and nanofat (Fig. 3e, f). In vitro culture of ADSCs showed that FE could promote ADSC proliferation in a dose-dependent manner (Fig. 6a). More importantly, cells that expanded in the presence of FE maintained their “stemness”, which was demonstrated by the consistent expression of cell surface markers, and retained their multipotent differentiation capacity at passage 5 (Fig. 6b, c). These results suggested that co-transplanted FE might stimulate the proliferation of surviving ADSCs in the grafts that would produce more adipogenic progenitors during the subsequent fat regeneration process. Cai et al. proposed that haematopoietic stem cells in the host could be mobilized and recruited to the site of transplanted fat to facilitate better survival [33]. Whether FE can recruit adipogenic progenitors to the graft and contribute to fat regeneration should be investigated in the future. In the current study, we injected a given volume of FE for each experiment. It is better to normalize the injection by measuring the amount of growth factors in each donor, because the composition of FE maybe different from individuals with different age, gender and body mass index. Although our previous study did not found a big difference between the samples from 6 donors using ELISA analysis [21], it is worth to be analysed with more samples in the future.

**Fig. 8:**
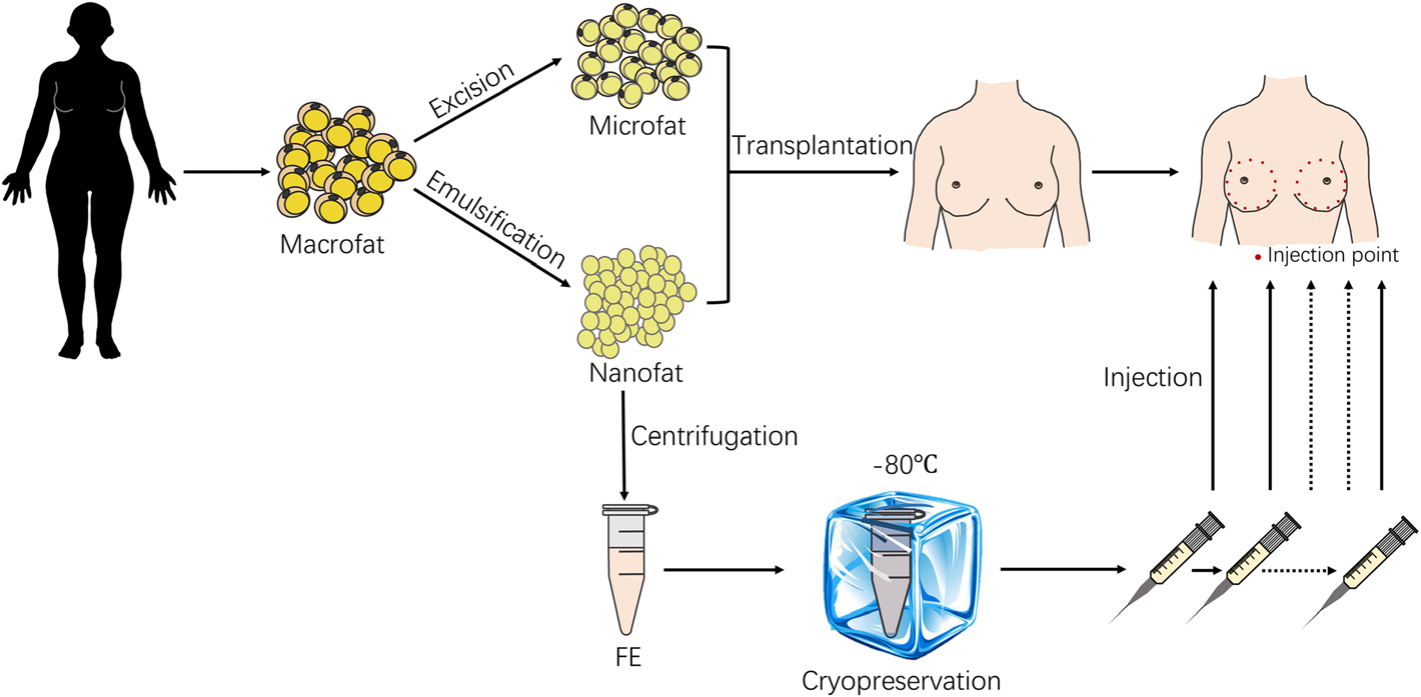
A new FE plus nanofat-assisted fat grafting strategy for future clinical application

Although nanofat showed a better improvement of fat survival than FE, it has several limitations because of its cellular component, including the difficulties of long-term storage for multiple administrations. In contrast, without cellular or oil fractions, FE can theoretically avoid cell-related concerns and undergo long-term storage more successfully. Taking advantage of nanofat and FE, we proposed a new strategy for fat grafting. Macrofat was first co-transplanted with nanofat, followed by 5 intra-graft injections of FE weekly post-transplantation. Better fat graft retention was observed in the nanofat+FE group than in the nanofat+PBS group (Fig. 7). These results prove that FE and nanofat synergistically improved fat graft survival. Therefore, for future clinical applications, if enough fat tissue could be aspirated from the donor site, part of the fat could be prepared into microfat for transplantation into the defects, part of the fat could be emulsified into nanofat and co-transplanted immediately as an assisting component, and the rest of the fat could be processed to obtain FE for subsequent intra-graft injections (Fig. 8). The microfat/nanofat/FE ratio in the graft should be optimized in future studies.

## Conclusions

The current study demonstrated that cell-free FE could improve fat graft survival via proangiogenic, anti-apoptotic and pro-proliferative effects on ADSCs. FE plus nanofat-assisted fat grafting is a new strategy that could potentially be used in clinical applications.

## Data Availability

The datasets generated during and/or analysed during the current study are available from the corresponding author on reasonable request.

## Abbreviations

ADSCs: Adipose-derived stem cells
BDNF: Brain-derived neurotrophic factor
bFGF: Basic fibroblast growth factor
DMEM: Dulbecco’s modified Eagle medium
EGF: Epidermal growth factor
FBS: Fetal bovine serum
FE: Fat extract
GM-CSF: Granulocyte-macrophage colony-stimulating factor
HGF: Hepatocyte growth factor
HUVECs: Human vascular endothelial cells
IGF-1: Insulin-like growth factor 1
PBS: Phosphate-buffered saline
PDGF: Platelet-derived growth factor
RNA: Ribonucleic acid
RNase: Ribonuclease
SVF: Stromal vascular fraction
TGF-β: Transforming growth factor beta
VEGF: Vascular endothelial growth factor
